# Inappropriate Detection of a Supraventricular Tachycardia as Dual Tachycardia by the PR Logic™ Algorithm

**DOI:** 10.1016/s0972-6292(16)30758-6

**Published:** 2014-05-25

**Authors:** Ajit Thachil, Sridevi Chennapragada, Narasimhan Calambur

**Affiliations:** 1 Lisie Hospital, Kochi, Kerala, India; 2 Care Hospital, Hyderabad, India

**Keywords:** ICD, PR logic, discriminators, dual tachycardia, inappropriate shock

## Abstract

Tachycardia detection and therapy algorithms in Implantable Cardioverter-Defibrillators (ICD) reduce, but do not eliminate inappropriate ICD shocks. Awareness of the pros and cons of a particular algorithm helps to predict its utility in specific situations. We report a case where PR logic™, an algorithm commonly used in currently implanted ICDs to differentiate supraventricular tachycardia (SVT) from ventricular tachycardia resulted in inappropriate detection and shock for an SVT, and discuss several solutions to the problem.

## Introduction

The PR logic™ algorithm, incorporated into all dual chamber Implantable Cardioverter-Defibrillators (ICD) and cardiac resynchronization therapy-defibrillators (CRTD) from Medtronic™ is commonly used in several currently implanted ICDs and CRTDs to differentiate supraventricular tachycardia (SVT) from ventricular tachycardia (VT). Though this algorithm has proven efficacy in tachycardia discrimination, there are specific situations where a fallacy in the algorithm can misclassify SVT as VT, as illustrated in the following case example.

## Case

**Figure 1:**
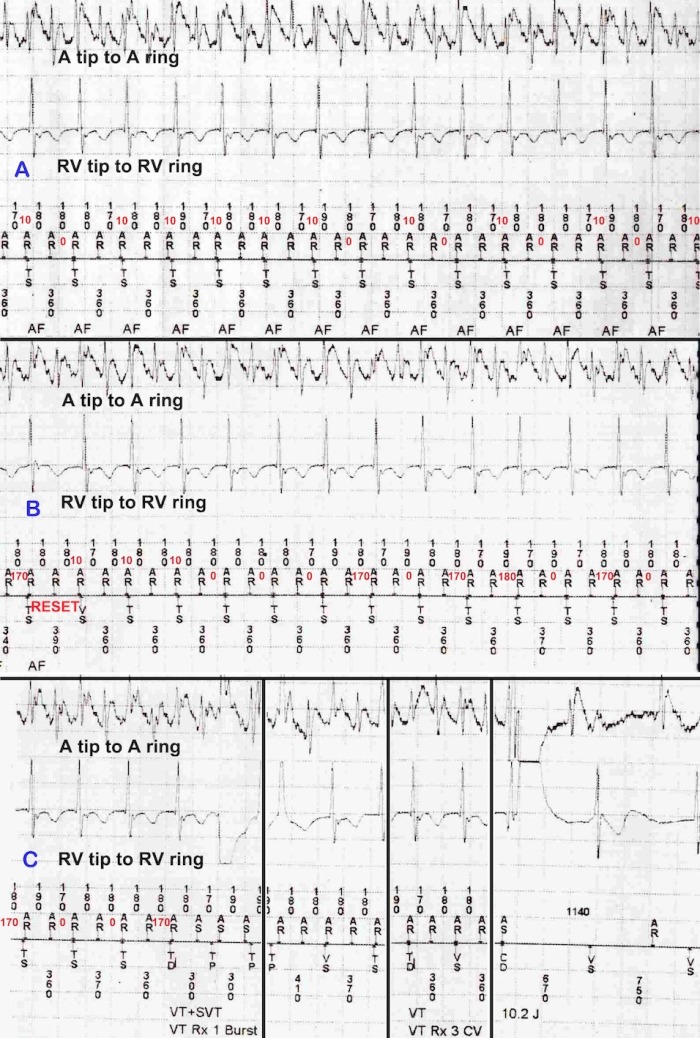
Initial appropriate, and subsequent inappropriate classification of the same SVT by the P-R logic™ algorithm. Device-recorded* P-R intervals are marked in red. Panel A shows detection of a 2:1 SVT. R-R intervals are regular. P-R intervals vary from 0-10 ms; no P-R interval is therefore different from the mean P-R interval by >40 ms (P-R association). Panel B (continuous with Panel A) shows a transient change in AV conduction resulting in a single beat with a PR interval of 170 ms and subsequent P-R intervals ranging from 0-180 ms. Within rolling windows of 8 P-R intervals, most P-R intervals now differ from the mean P-R interval by > 40 ms (P-R dissociation). Transient change in AV conduction also results in a single Vs beat (Panel B), resetting the NID counter. The same tachycardia is now reclassified as SVT+VT, leading to inappropriate therapies. Panel C (continuous with Panel B; vertical lines indicate discontinuity in EGM within Panel C) depicts unsuccessful ATPs followed by ICD shock terminating the SVT. * These PR intervals are not displayed by the device, and have been manually derived from the A-A and V-V intervals in the recorded electrogram.

A 60 year old male with presyncope and severe left ventricular systolic dysfunction due to ischemic cardiomyopathy received a Medtronic Maximo DR 7278 dual chamber ICD. VT detection and therapy were programmed as follows: VT zone: 155 beats/min (387 ms), number of intervals to detect (NID): 16; VT therapies: Anti-tachycardia pacing (ATP) X 2, followed by cardioversion. Four years after ICD implantation, the patient presented with recurrent ICD shocks. ICD interrogation showed a tachycardia which was initially detected appropriately as SVT, but was subsequently reclassified wrongly as SVT + VT (dual tachycardia) resulting in inappropriate ICD shock (Figure 1). The patient underwent an electrophysiology study during which right atrial flutter was induced and ablated. Post-ablation, he has been followed up for over two years without ventricular arrhythmia or ICD therapies. 

## Discussion

A sudden onset stable tachycardia in the VT zone, with two P-P intervals per R-R interval where the P-P intervals do not satisfy far-field R wave criteria is diagnosed as SVT by the P-R logic™ algorithm (Figure 1, Panel A) [1]. A single V-sense interval during an ongoing SVT resets the NID counter without reapplying the onset criterion as occurred in this example, whereas ≥ 8 consecutive V-senses cause reversion to onset for re-detection (Figure 1, Panel B). P-R logic™ continuously looks for evidence of R-R regularity and P-R dissociation; if both of these are satisfied by a tachycardia satisfying the SVT criteria, a diagnosis of SVT+VT (dual tachycardia) is made (Figure 1, Panel C). For consideration of R-R regularity, rolling windows of 18 beats each are continuously analysed. The number of R-R intervals in the two largest 10 ms bins within each 18-beat rolling window is divided by 18; if this ratio ("modesum ratio") is ≥14/18, R-R regularity is diagnosed. P-R dissociation is sought for in a rolling window of 8 beats. P-R intervals are rounded down to the nearest 10 ms, and the mean P-R interval within each rolling window of 8 P-Rs is calculated. If an individual P-R interval differs from the mean by ≥ 40 ms, or if an R-R interval contains no Ps, that interval is declared dissociated; ≥ 4 dissociated intervals within the window of 8 constitutes P-R dissociation [1]. P-R interval detection by this algorithm has two fallacies that caused the inappropriate detection in this case - i) Simultaneous occurrence of P and R is allocated a P-R interval of 0 ms. ii) When two P waves fall within an R-R interval, the P wave just preceding the R is always considered for calculation of the P-R interval, allowing for non-physiological short P-R intervals such as 10 or 20 ms. Alternate P waves often occur simultaneously with or just before or just after R waves in regular SVTs with 2:1 atrioventricular conduction, setting the stage for the above fallacies.

During regular SVTs with 2:1 atrioventricular conduction, each R-R interval often contains two P waves. In this scenario, it is often the first P wave that is conducted to the ventricle. This is in contrast to the assumption made by the P-R logic™ algorithm, which uses the P wave just preceding the R or coinciding with the R wave for calculation of the P-R interval. The algorithm thus allows non-physiological short P-R intervals such as 0, 10 or 20 ms. Incorporation of a lower rejection limit for P-R intervals to the existing P-R logic™ algorithm can avoid this fallacy.

Addition of morphology matching (the Wavelet™ function) to the discrimination sequence (available in the newer Protecta™, Brava™ and Viva™ family of devices from Medtronic™) can reduce inappropriate detection as occurred in this case. However, a significant proportion of currently implanted dual chamber ICD and CRTD devices belong to the previous generation series (Entrust DR™, Maximo DR™, Marquis DR™, InSync Maximo™, InSync Marquis™, Maximo II DR™, Secura DR™ and Consulta CRTD™) from Medtronic™, which lack the Wavelet™ function. Even in devices employing the Wavelet™ function, relying on Wavelet™ as the key discrimination criterion (as would be the case with such a sudden onset stable SVT with regular 2:1 atrioventricular conduction) tends to cause inappropriate detection of SVT as VT. In the WAVE study, which prospectively evaluated Wavelet™ as the sole criterion to discriminate SVT from VT, 39.7% of 885 spontaneous SVT episodes were detected as VT by the Wavelet algorithm [2].

Ventricular rates during SVT with regular 2:1 atrioventricular conduction usually do not exceed 170-180 bpm. Programming the VT zone to 180 bpm (330 ms) could have avoided the inappropriate detection; recent recommendations also support such programming of ICDs to treat only the more rapid ventricular rhythms, unlike the default programming of the device [3-4]. In certain situations, it may be clinically deemed necessary to program the VT detection limit to relatively lower rates. In such situations, AV nodal slowing therapies to ensure at least intermittent 3:1 atrioventricular block during SVT can help to avoid this scenario by causing irregular ventricular rate (in intermittent 3:1 atrioventricular block) or ventricular rates slower than the VT detection rate (in persistent 3:1 atrioventricular block).

## Conclusion

The PR logic™ algorithm can misdiagnose sudden onset regular SVT with stable 2:1 atrioventricular conduction as VT due to assumption of non-physiologically short PR intervals. In addition to incorporating a Wavelet function in the discrimination sequence, programming a faster VT detection limit or ensuring >2:1 atrioventricular block during SVT with AV nodal blocking drugs may be required to prevent inappropriate detection and therapy.
